# Adherence to diabetes risk reduction diet and the risk of head and neck cancer: a prospective study of 101,755 American adults

**DOI:** 10.3389/fnut.2023.1218632

**Published:** 2023-09-22

**Authors:** Xia Wu, Linglong Peng, Haoyun Luo, Zhiquan Xu, Jijian Wang, Haitao Gu, Yaxu Wang, Yi Xiao, Chaohua Zhang, Ling Xiang

**Affiliations:** ^1^Department of Health Management Centre, Chongqing General Hospital, Chongqing, China; ^2^Department of Gastrointestinal Surgery, The Second Affiliated Hospital of Chongqing Medical University, Chongqing, China; ^3^Department of Clinical Nutrition, The Second Affiliated Hospital of Chongqing Medical University, Chongqing, China

**Keywords:** diabetes risk reduction diet, head and neck cancer, epidemiology, cohort study, diet

## Abstract

**Background:**

Adherence to the diabetes risk reduction diet (DRRD) may potentially reduce the risk of developing head and neck cancer (HNC) as the diet includes fruits and limits red and processed meats, known risk factors for HNC. However, there is currently no epidemiological research to investigate this potential association.

**Methods:**

The present study utilized data on demographics, lifestyles, medications, and diets of participants from the Prostate, Lung, Colorectal, and Ovarian (PLCO) Cancer Screening Trial to explore the potential association between adherence to DRRD and the risk of HNC. We used a DRRD score to evaluate adherence to the dietary pattern and employed Cox regression analysis to calculate hazard ratios (HRs) and 95% confidence intervals (CIs) for HNC risk. Several subgroup analyses were carried out to identify potential effect modifiers, and multiple sensitivity analyses were performed to evaluate the stability of the correlation. The nine components of the DRRD was assessed separately for its association with the risk of HNC.

**Results:**

During a mean follow up of 8.84 years, 279 cases of HNC were observed. DDRD score was found to be inversely associated with the risk of HNC (HR _Q4 vs. Q1_: 0.582; 95% CI: 0.396, 0.856; *p* = 0.005 for trend) in a linear dose–response manner (*p* = 0.211 for non-linearity). Subgroup analysis indicated this inverse correlation was more pronounced among participants who had never smoked (HR_Q4 vs. Q1_: 0.193; 95% CI: 0.073, 0.511; *p* < 0.001 for trend) compared to current or former smokers (*p* = 0.044 for interaction). The primary association of DDRD and HNC risk remained robust after several sensitivity analyses. Regarding the individual components of DRRD, an inverse association was also observed between the risk of HNC and increased intake of cereal fiber and whole fruit (all *p* < 0.05 for trend).

**Conclusion:**

Our findings provide evidence that following the DRRD pattern may reduce the risk of NHC, especially for non-smokers.

## Introduction

Head and neck cancer (HNC) is a prevalent type of cancer, ranking as the seventh most common globally (1). In the United States, 53,000 new cases of HNC and 10,860 deaths caused by HNC were reported in 2019 (2). Numerous studies have consistently shown that exposure to smoking and alcohol, poor oral hygiene, infection with Epstein–Barr virus (EBV) or human papillomavirus (HPV), as well as exposure to certain chemicals or radiation, are established as primary risk factors for HNC (3, 4). Recent researches in the field of HNC has highlighted the potential influence of dietary factors on the development of HNC (5). A diet rich in fruits and vegetables may be associated with a decreased risk of developing HNC (6), while high intake of red and processed meats may increase the risk of HNC (5). However, it should be emphasized that assessing the influence of singular foods or nutrients on tumor susceptibility may not provide a comprehensive understanding of the impact of dietary intake as a whole.

The diabetes risk reduction diet (DRRD) has gained popularity as a dietary pattern designed to prevent and control diabetes (7). The DRRD emphasizes a high proportion of cereal fiber, coffee, nuts, whole fruits, and a ratio of polyunsaturated to saturated fat, while limiting trans-fat, glycemic index (GI), sugar-sweetened beverages (SSBs), and red and processed meats (8). Since the DRRD dietary pattern includes a high intake of fruits and limits red and processed meats, it is possible that adhering to DRRD may reduce the risk of developing HNC. Additionally, although originally developed for diabetes prevention, studies have shown that following the DRRD may also reduce the incidence of several types of cancer, including lung (9), endometrial (10), breast (8), and pancreatic (11) cancers. Furthermore, the increased susceptibility of people with diabetes to HNC (12) further supports that adherence to the DRRD may have a potential link to reduced risk of HNC. However, there is currently limited research on this potential association.

To address this gap, we performed a prospective study to clarify the association of DRRD dietary pattern and the risk of HNC in a large American population. By conducting this prospective designed analysis, we aim to gain a better understanding of the potential role of the DRRD in preventing HNC, and to provide more comprehensive dietary recommendations to the public for reducing the risk of HNC.

## Materials and methods

### Study design and population

This study utilized data from the Prostate, Lung, Colorectal, and Ovarian (PLCO) Cancer Screening Trial. The PLCO trial is a large randomized controlled trial that was designed to evaluate the effectiveness of cancer screening tests for reducing cancer mortality rates. This trial was conducted between 1993 and 2001 at 10 clinical centers in the United States and enrolled 154,887 participants aged 55–74. All participants were randomly assigned to either a control group or an intervention group involving screening tests. The follow-up period extended until 2009 for the incidence of over 20 types of cancer, including HNC, and until 2018 for cancer-related mortality. The PLCO trial extensively collected data on the demographic characteristics, health history, lifestyle factors, and diet information of the participants through self-reported questionnaires. In this trial, participants were asked to complete two questionnaires: the baseline questionnaire (BQ) and the diet history questionnaire (DHQ) at the beginning of the trial. The DHQ relied on a 137-item food frequency questionnaire (FFQ) to gather data on dietary information over the past year, and the DRRD dietary pattern can be well established using the dietary data collected through DHQ. Detailed information on the PLCO trial has been reported in related literature (13).

**Figure 1:**
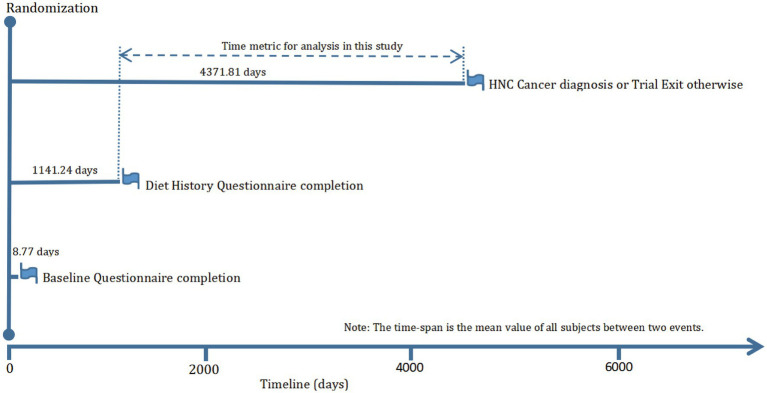
The timeline and follow-up scheme of our study.

**Figure 2:**
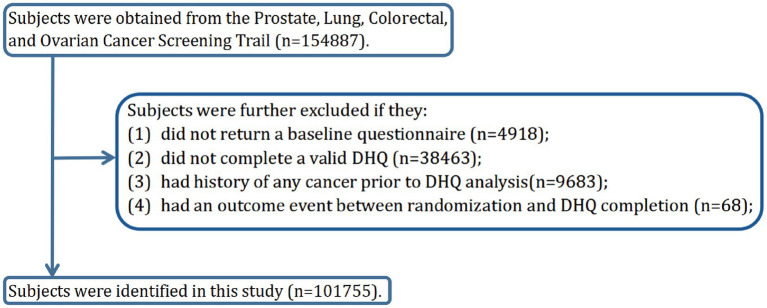
The flow chart of identifying eligible participants. PLCO, Prostate, Lung, Colorectal, and Ovarian; BQ, baseline questionnaire; DHQ, diet history questionnaire.

The objective of our current study was to investigate whether adherence to the DRRD is related to the risk of HNC. The primary endpoint was defined as the diagnosis of HNC among participants, and the follow-up time was determined as the period from DHQ completion to the occurrence of HNC, death, loss during follow-up, or the end of the follow-up period (i.e., December 31, 2009), whichever occurred first (Figure 1). To achieve the study objective, a set of exclusion criteria were employed to establish an appropriate study cohort from an initial pool of 154,887 participants. Firstly, 4,918 participants who did not return the BQ were excluded. Secondly, 38,463 participants who either did not return the DHQ or returned an incomplete DHQ that having at least 8 missing frequency responses of dietary items, a missing completion date, completion date after death, or extreme energy consumption (top 1% and bottom 1%) were excluded. Thirdly, 9,683 participants with a history of any cancer prior to DHQ entry were excluded. Fourthly, 68 participants who experienced an outcome event between DHQ entry and DHQ completion were excluded. Ultimately, the remaining cohort comprised 101,755 individuals in our study, as illustrated in Figure 2.

### Assessment of DRRD dietary pattern

To evaluate the adherence of each participant to DRRD, a DRRD score was calculated based on the methodology described in previous studies (9). Briefly, the intakes of the nine DRRD components were obtained from the DHQ, and then each component was categorized into five groups based on its quintile values of dietary intake, and assigned scores ranging from 1 to 5. For cereal fiber, coffee, nuts, whole fruits, and the ratio of polyunsaturated to saturated fat, a higher quintile value indicated a higher score. Conversely, for trans-fat, GI, SSBs, and red and processed meats, a lower quintile value indicated a higher score. The DRRD score was then calculated by summing the scores of the nine components, resulting in a range of 9 to 45. An increased DRRD score indicates greater adherence to the DRRD dietary pattern. Detailed data for determining DRRD score was shown in Supplementary Table S1.

### HNC ascertainment

The ascertainment of HNC cases primarily relied on the administration of an annual study update form, which was disseminated by each screening center to participants. This form was designed to elicit information on whether individuals had received a diagnosis of HNC, along with the date and location of the diagnosis, and the contact details of their healthcare providers. HNC cases were defined based on the following ICD-O-2 codes for malignant tumors: (1) oral cavity: C00.3–C00.9, C02.0–C02.3, C03.0, C03.1, C03.9, C04.0, C04.1, C04.8, C04.9, C05.0, C06.0–C06.2, C06.8 and C06.9; (2) oropharynx: C01.9, C02.4, C05.1, C05.2, C09.0, C09.1, C09.8, C09.9, C10.0, C10.2–C10.4, C10.8 and C10.9; (3) hypopharynx: C12.9, C13.0–C13.2, C13.8 and C13.9; (4) oral cavity or pharynx NOS: C02.8, C02.9, C05.8, C05.9, C14.0, C14.2 and C14.8; and (5) larynx: C10.1, C32.0–C32.3 and C32.8–C32.9. Cases of NHC reported through this form were subjected to further verification by scrutinizing any available medical records. In addition, supplementary sources such as death certificates and family reports were utilized to augment the ascertainment process. To ensure consistency in case selection, only participants who had received a diagnosis of HNC were included in the study.

### Assessment of covariates

The study gathered information on age at DHQ completion, drinking status and alcohol consumption, energy intake, food and nutrient consumption via the DHQ. Daily food intake was determined by multiplying food frequency by portion size, while daily nutrient intake was estimated using the USDA’s 1994–96 Continuing Survey of Food Intakes by Individuals and the Nutrition Data Systems for Research (14). The detailed calculation methods of dietary fiber, GI, and trans-fat are available in previously published literatures (15–17). Additional data or covariates, such as gender, race, body mass index (BMI), educational level, marital status, smoking status, pack-years of smoking, history of diabetes, and family history of HNC, were obtained using a self-administered baseline questionnaire.

### Statistical analysis

To reduce potential biases and enhance the statistical power of our study, imputation was performed using modal values for categorical variables and median values for continuous variables. Supplementary Table S2 displays the distribution of variables with missing values before and after imputation. To examine the potential impact of data imputation on our results, we also repeated the primary statistical analyses in the population with complete covariate data in the subsequent sensitivity analysis.

To evaluate the association between DRRD and HNC risk, the study employed Cox proportional hazards regression as the primary analysis model, with follow-up period as the time metric. The DRRD score was categorized into quartiles, with the lower quartile serving as the reference group. Person-years of each quartile were estimated based on the duration of follow-up. To assess whether a linear trend could be observed across quartiles of DRRD scores for estimating HNC risk, median values of each quartile were assigned to individuals within the corresponding quartile and treated as a continuous variable in regression models. After examining the Schoenfeld residuals, we found that the proportional hazards assumption of the Cox regression model was satisfied (P for global test > 0.05). Multivariable regression models were utilized to further adjust potential covariates. Specifically, model 1 was adjusted for age, sex, and race. Model 2 included additional adjustments for marital status, educational level, BMI, family history of HNC, smoking status, pack-years of smoking, drinking status, alcohol consumption, history of diabetes, and energy from diet. A restricted cubic spline model with three knots (i.e., 10th, 50th, and 90th percentiles of DRRD score) was employed to analyze HNC risk across the entire range of the DRRD score. Additionally, we conducted further analyses to investigate the association between the nine dietary components of the DRRD and HNC risk using similar Cox regression model as described above. Specifically, we obtained the intake of each dietary component of the DRRD from the DHQ and divided them into quartiles, with the lowest quartile serving as the reference group.

To investigate whether the association between DRRD score and HNC risk was modified by various factors, subgroup analyses were conducted. Participants were divided into categories based on age (>65 vs. ≤65 years), sex (male vs. female), BMI (≤25 vs. >25 kg/m2), smoking status (never vs. current or former), pack-years of smoking (≤medium vs. >medium), drinking status (no vs. yes), alcohol consumption (≤medium vs. >medium), history of diabetes (no vs. yes), Family history of HNC (no vs. yes), and energy from diet (≤medium vs. >medium). Interaction *p* values were computed by comparing models with and without multiplicative interaction terms before subgroup analyses to avoid spurious subgroup effects. Additionally, sensitivity analyses were conducted to confirm the robustness of the primary results. These included repeating the primary analysis in participants with complete data, excluding participants with diabetes, excluding participants with follow-up less than 2 years, excluding participants with extreme energy intake (>4,000 kcal/day or <500 kcal/day), and excluding participants with extreme BMI (top 1% and bottom 1%).

The statistical significance level was set at a *p* value of < 0.05. R 4.2.1 software was utilized for all statistical analyses.

## Results

### Baseline characteristics

In this study, a total of 101,755 individuals were included and categorized into quartiles based on their DRRD scores: quartile 1 (*n* = 27,890) with scores between 9 and 23, quartile 2 (*n* = 28,970) with scores between 24 and 27, quartile 3 (*n* = 19,784) with scores between 28 and 30, and quartile 4 (*n* = 25,111) with scores between 31 and 45. The mean (standard deviation) DRRD score for all participants was 26.84 (5.31), and their baseline characteristics were presented in Table 1. Compared to the lowest quartile group, individuals in the highest quartile group tended to be female, older, have a lower BMI, non-smoker or have fewer pack-years of smoking, a drinker or have high alcohol consumption, and have no history of diabetes. Moreover, those in the highest quartile of DRRD scores had a lower intake of energy compared to those in the lowest quartile.

**Table 1:** Baseline characteristics of study population according to overall diabetes risk reduction diet score.

Characteristics	Overall	Quartiles of diabetes risk reduction diet scores				*p*-value
		Quartile 1 (9–23)	Quartile 2 (24–27)	Quartile 3 (28–30)	Quartile 4 (31–45)	
Number of participants	101,755	27,890	28,970	19,784	25,111	
Diabetes risk reduction diet score	26.84 ± 5.31	20.43 ± 2.37	25.53 ± 1.11	28.93 ± 0.81	33.80 ± 2.56	0.000
Age (years)	65.53 ± 5.73	64.66 ± 5.61	65.54 ± 5.71	65.90 ± 5.77	66.18 ± 5.75	<0.001
**Sex**						0.000
Male	49,496 (48.6%)	16,282 (58.4%)	14,856 (51.3%)	8,839 (44.7%)	9,519 (37.9%)	
Female	52,259 (51.4%)	11,608 (41.6%)	14,114 (48.7%)	10,945 (55.3%)	15,592 (62.1%)	
**Race**						0.899
Non-hispanic	100,136 (98.41%)	27,449 (98.42%)	28,497 (98.37%)	19,478 (98.45%)	24,712 (98.41%)	
Hispanic	1,619 (1.59%)	441 (1.58%)	473 (1.63%)	306 (1.55%)	399 (1.59%)	
**Marital status**						<0.001
Live together	79,826 (78.45%)	22,143 (79.39%)	23,066 (79.62%)	15,509 (78.39%)	19,108 (76.09%)	
Live alone	21,929 (21.55%)	5,747 (20.61%)	5,904 (20.38%)	4,275 (21.61%)	6,003 (23.91%)	
**Education level**						<0.001
College below	42,937 (42.20%)	13,845 (49.64%)	12,756 (44.03%)	7,850 (39.68%)	8,486 (33.79%)	
College and beyond	58,818 (57.80%)	14,045 (50.36%)	16,214 (55.97%)	11,934 (60.32%)	16,625 (66.21%)	
Body mass index (kg/m^2^)	27.2 ± 4.8	28.2 ± 5.0	27.5 ± 4.7	26.9 ± 4.6	26.0 ± 4.4	0.000
**Smoking status**						<0.001
Never	48,580 (47.74%)	12,409 (44.49%)	13,572 (46.85%)	9,700 (49.03%)	12,899 (51.37%)	
Current	9,401 (9.24%)	3,772 (13.52%)	2,841 (9.81%)	1,488 (7.52%)	1,300 (5.18%)	
Former	43,774 (43.02%)	11,709 (41.98%)	12,557 (43.34%)	8,596 (43.45%)	10,912 (43.46%)	
Pack-years of smoking	17.65 ± 26.59	21.68 ± 30.02	18.37 ± 26.98	16.13 ± 25.02	13.55 ± 22.23	<0.001
**Drinking status**						< 0.001
No	27,757 (27.28%)	8,654 (31.03%)	7,780 (26.86%)	5,006 (25.30%)	6,317 (25.16%)	
Yes	73,998 (72.72%)	19,236 (68.97%)	21,190 (73.14%)	14,778 (74.70%)	18,794 (74.84%)	
Alcohol consumption (g/day)	9.53 ± 25.25	8.38 ± 23.51	9.69 ± 24.36	10.66 ± 28.09	9.75 ± 25.71	<0.001
**History of diabetes**						<0.001
No	94,949 (93.31%)	25,675 (92.06%)	26,957 (93.05%)	18,531 (93.67%)	23,786 (94.72%)	
Yes	6,806 (6.69%)	2,215 (7.94%)	2013 (6.95%)	1,253 (6.33%)	1,325 (5.28%)	
**Family history of head and neck cancer**						0.955
No	100,308 (98.58%)	27,490 (98.57%)	28,563 (98.60%)	19,496 (98.54%)	24,759 (98.60%)	
Yes	1,447 (1.42%)	400 (1.43%)	407 (1.40%)	288 (1.46%)	352 (1.40%)	
Energy from diet (kcal/day)	1738.63 ± 736.43	1796.97 ± 740.65	1739.26 ± 782.37	1715.34 ± 763.68	1691.48 ± 645.23	< 0.001
**Diabetes risk reduction diet components**						
Cereal fiber (g/day)	11.85 ± 5.70	9.32 ± 4.17	11.08 ± 5.15	12.49 ± 5.61	15.06 ± 6.20	0.000
Whole fruit (servings/day)	2.73 ± 2.04	1.68 ± 1.26	2.43 ± 1.73	3.03 ± 1.96	4.02 ± 2.38	0.000
Nuts (g/day)	6.73 ± 14.53	2.74 ± 5.68	4.95 ± 9.88	7.41 ± 14.83	12.68 ± 21.83	0.000
Coffee (g/day)	846.37 ± 794.46	730.20 ± 788.94	869.56 ± 802.57	892.66 ± 790.48	912.15 ± 780.59	<0.001
Ratio of polyunsaturated to saturated fat	0.76 ± 0.26	0.62 ± 0.18	0.72 ± 0.22	0.80 ± 0.24	0.95 ± 0.28	0.000
Glycemic index from diet	53.55 ± 3.31	55.64 ± 2.99	53.89 ± 3.01	52.78 ± 2.92	51.45 ± 2.74	0.000
Trans fat (g/day)	3.99 ± 2.39	4.94 ± 2.54	4.21 ± 2.50	3.67 ± 2.23	2.92 ± 1.65	0.000
Sugar-sweetened beverage (g/day)	264.50 ± 433.29	449.55 ± 565.48	264.39 ± 407.34	191.50 ± 329.20	116.61 ± 254.42	0.000
Red and processed meat (g/day)	12.42 ± 15.31	19.51 ± 19.26	13.41 ± 15.00	9.79 ± 11.83	5.46 ± 7.57	0.000

Values are means (standard deviation) for continuous variables and percentages for categorical variables.

### Association between DRRD score and HNC risk

**Figure 3:**
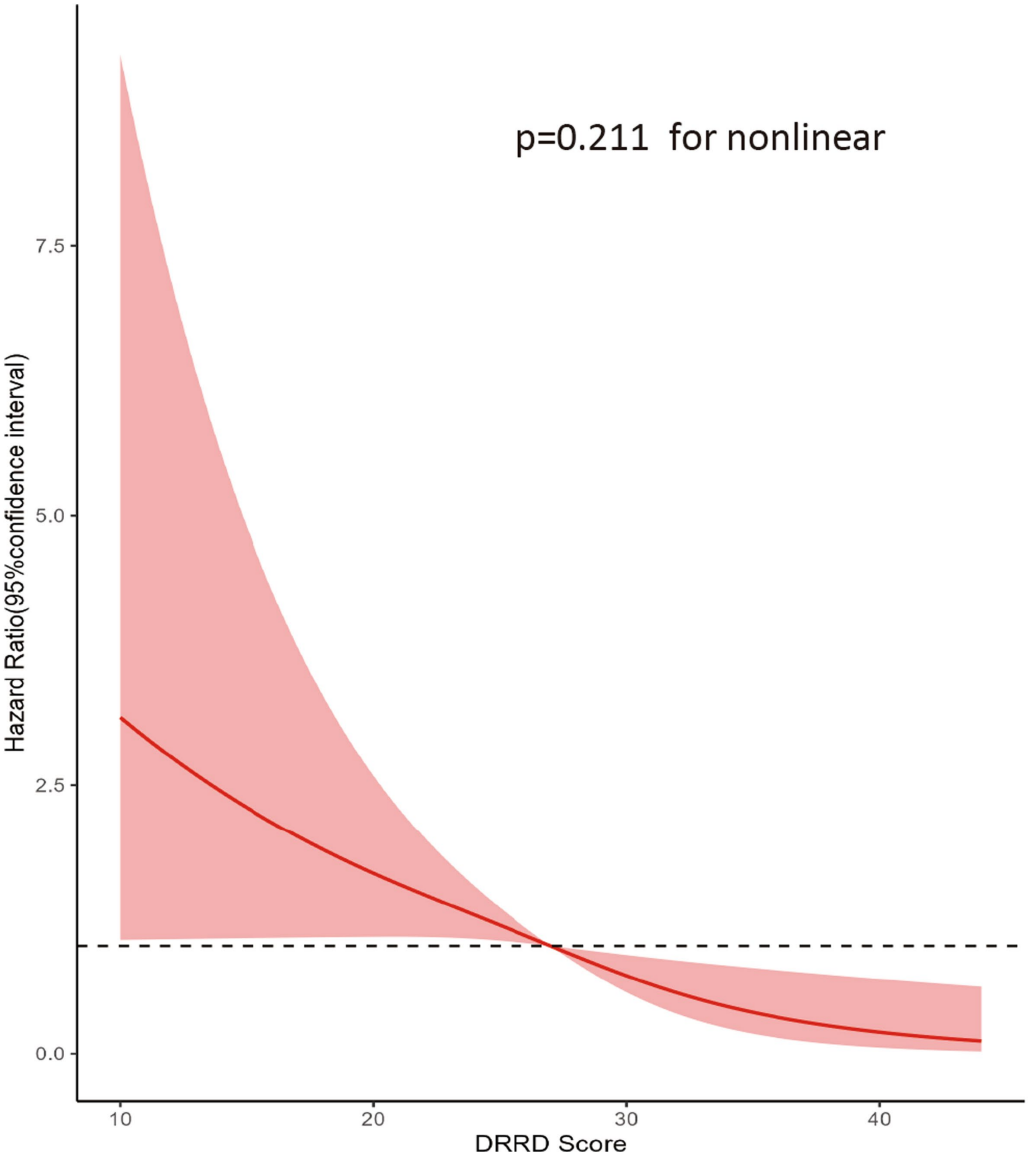
Dose-response association between DRRD score and the risk of HNC. Hazard ratio was adjusted for age, sex, marital status, race, educational level, BMI, family history of HNC, smoking status, pack-years of smoking, drinking status, alcohol consumption, history of diabetes, and energy from diet.

This study followed up with a total of 900001.9 person-years and recorded 279 cases of malignant primary HNC. The overall incidence was 3.1 cases/10,000 person-years with a mean (standard deviation) follow-up duration of 8.84 (1.92) years. The results of Cox regression analysis of the entire study population are presented in Table 2. The unadjusted model analysis showed that individuals in the highest quartile group had a lower risk of HNC compared to those in the lowest quartile group (HR_Q4 vs. Q1_: 0.392; 95% CI: 0.271, 0.568; *p* < 0.001 for trend). After adjusting for all potential confounding factors, the inverse association between DRRD score and HNC risk remained significant (HR_Q4 vs. Q1_: 0.582; 95% CI: 0.396, 0.856; *p* = 0.005 for trend). In the restricted cubic spline regression model, DRRD score was found to have an inverse association with the risk of HNC in a linear dose-response manner (*p* = 0.211 for non-linearity), as shown in Figure 3.

**Table 2:** Hazard ratios of the association of DRRD score with the risk of HNC.

Quartiles of DRRD score	Number of cases	Person-years	Incidence rate per 100 person-years (95% confidence interval)	Hazard ratio (95% confidence interval)		
				Unadjusted	Model 1^a^	Model 2^b^
Quartile 1 (9–23)	105	243469.8	0.043 (0.036, 0.052)	1.000 (reference)	1.000 (reference)	1.000 (reference)
Quartile 2 (24–27)	91	256387.8	0.035 (0.029, 0.044)	0.822 (0.621, 1.088)	0.874 (0.659, 1.158)	0.951 (0.716, 1.264)
Quartile 3 (28–30)	45	175735.3	0.026 (0.019, 0.034)	0.593 (0.418, 0.840)	0.678 (0.477, 0.964)	0.761 (0.533, 1.087)
Quartile 4 (31–45)	38	224409.0	0.017 (0.012, 0.023)	0.392 (0.271, 0.568)	0.486 (0.334, 0.707)	0.582 (0.396, 0.856)
*P* for trend				**<0.001**	**<0.001**	**0.005**

a Adjusted for age, sex and race.

b Adjusted for model 1 plus marital status, educational level, BMI, smoking status, pack-years of smoking, drinking status, alcohol consumption, history of diabetes, family history of HNC, and energy from diet. The bold values are indicate statistical significance, with *p*-values less than 0.05.

### Subgroup analyses

The subgroup analyses results are presented in Table 3, indicating that the inverse correlation between DRRD score and HNC risk was not modified by various factors such as age, sex, BMI, pack-years of smoking, drinking status, alcohol consumption, history of diabetes and energy from diet (all *P* for interaction > 0.05). However, the inverse correlation was more pronounced among participants who had never smoked (HR_Q4 vs. Q1_: 0.193; 95% CI: 0.073, 0.511; *p* < 0.001 for trend) compared to current or former smokers (*p* = 0.044 for interaction).

**Table 3:** Subgroup analyses on the association of DRRD score with the risk of HNC.

Subgroup variable	No. ofcases	Person-years	Hazard ratio (95% confidence interval) by DRRD score				*P*_trend_	*P*_interaction_
			Quartile 1	Quartile 2	Quartile 3	Quartile 4		
**Age**								0.331
≤65 years old	128	469864.9	1.00 (reference)	0.706 (0.462, 1.079)	0.613 (0.359, 1.046)	0.518 (0.294, 0.912)	0.008	
>65 years old	151	430137.0	1.00 (reference)	1.129 (0.762, 1.673)	0.804 (0.496, 1.303)	0.534 (0.316, 0.901)	0.016	
**Sex**								0.795
Male	222	432707.9	1.00 (reference)	0.966 (0.707, 1.321)	0.753 (0.505, 1.121)	0.532 (0.34, 0.832)	0.004	
Female	57	467294.0	1.00 (reference)	0.656 (0.335, 1.284)	0.518 (0.238, 1.129)	0.454 (0.215, 0.957)	0.028	
**Body mass index (kg/m** ^**2**^ **)**								0.43
≤25	97	307723.0	1.00 (reference)	0.677 (0.404, 1.134)	0.611 (0.335, 1.112)	0.557 (0.311, 0.997)	0.038	
>25	182	592278.9	1.00 (reference)	1.060 (0.753, 1.491)	0.793 (0.511, 1.231)	0.521 (0.311, 0.874)	0.018	
**Smoking status**								**0.044**
Never	63	436330.0	1.00 (reference)	0.686 (0.387, 1.216)	0.426 (0.199, 0.914)	0.193 (0.073, 0.511)	**<0.001**	
Current or former	216	463671.8	1.00 (reference)	1.007 (0.726, 1.397)	0.831 (0.556, 1.243)	0.687 (0.451, 1.046)	0.076	
**Pack-years of smoking**								0.069
≤medium	67	462817.4	1.00 (reference)	0.672 (0.386, 1.172)	0.444 (0.214, 0.918)	0.181 (0.069, 0.475)	<0.001	
>medium	212	437184.4	1.00 (reference)	0.962 (0.692, 1.337)	0.757 (0.504, 1.136)	0.613 (0.403, 0.932)	0.017	
**Drinking status**								0.393
No	62	243394.2	1.00 (reference)	1.056 (0.6, 1.858)	0.403 (0.154, 1.051)	0.487 (0.209, 1.135)	0.042	
Yes	217	656607.6	1.00 (reference)	0.878 (0.633, 1.218)	0.773 (0.525, 1.14)	0.521 (0.339, 0.801)	0.003	
**Alcohol consumption (g/day)**								0.549
≤medium	99	453246.6	1.00 (reference)	0.876 (0.554, 1.385)	0.507 (0.26, 0.989)	0.422 (0.214, 0.83)	0.005	
>medium	180	446755.2	1.00 (reference)	0.954 (0.664, 1.371)	0.848 (0.553, 1.3)	0.599 (0.375, 0.958)	0.039	
**History of diabetes**								0.582
No	262	843913.7	1.00 (reference)	0.911 (0.68, 1.222)	0.738 (0.514, 1.06)	0.508 (0.341, 0.756)	0.001	
Yes	17	56088.0	1.00 (reference)	0.913 (0.301, 2.764)	0.24 (0.029, 1.98)	0.692 (0.172, 2.79)	0.362	
**Energy from diet (kcal/day)**								0.375
≤medium	99	450318.8	1.00 (reference)	1.093 (0.684, 1.747)	0.546 (0.285, 1.048)	0.509 (0.267, 0.968)	0.016	
>medium	180	449683.0	1.00 (reference)	0.809 (0.564, 1.159)	0.793 (0.519, 1.211)	0.522 (0.324, 0.841)	0.009	

Hazard ratios were adjusted for age, sex, race, marital status, educational level, BMI, smoking status, pack-years of smoking, drinking status, alcohol consumption, history of diabetes, family history of HNC, and energy from diet.

The bold values are indicate statistical significance, with *p*-values less than 0.05.

### Sensitivity analyses

The results of several sensitivity analyses are presented in Table 4, and indicate that the inverse association between DRRD score and HNC risk did not change significantly, thereby further confirming the robustness of our primary findings. Specifically, when Cox regression analyses were repeated in participants with complete covariate data, we obtained similar results (HR_Q4 vs. Q1_: 0.542; 95% CI: 0.360, 0.816; *p* = 0.003 for trend).

**Table 4:** Sensitivity analyses on the association of DRRD score with the risk of HNC.

Categories	HR _Quartile 4 vs. Quartile 1_ (95% CI)^a^	*P* for trend
Repeating analysis in participants with complete data^b^	0.542 (0.360, 0.816)	**0.003**
Excluding participants with diabetes^c^	0.580 (0.388, 0.865)	**0.009**
Excluding participants with a follow-up less than 2 years	0.587 (0.383, 0.901)	**0.015**
Excluding participants with extreme energy intake^d^	0.551 (0.372, 0.816)	**0.002**
Excluding participants with extreme BMI^e^	0.581 (0.394, 0.858)	**0.005**

a HR was adjusted for age, sex, race, marital status, educational level, BMI, smoking status, pack-years of smoking, drinking status, alcohol consumption, history of diabetes, family history of HNC, and energy from diet.

b Sample size of participants with complete data: *n* = 98,037.

c HR was not adjusted for history of diabetes.

d The extreme energy intake was defined as >4,000 kcal/day or <500 kcal/day.

e The extreme BMI was defined as top 1% or bottom 1% in the included population. The bold values are indicate statistical significance, with *p*-values less than 0.05.

### Dietary components of DRRD and the risk of HNC

We further investigated the association between each dietary components of DRRD and the risk of HNC. Our Results indicated that individuals in the highest quartile of cereal fiber and whole fruit consumption, considered as “favorable” DRRD components, had a lower risk of HNC compared to those in the lowest quartile [(cereal fiber: HR_Q4 vs. Q1_: 0.471; 95% CI: 0.301, 0.739; *p* = 0.002 for trend) (Supplementary Table S3) and (whole fruit: HR _Q4 vs. Q1_: 0.555; 95% CI: 0.372, 0.829; *p* = 0.002 for trend) (Supplementary Table S4)]. However, there was no significant association between the risk of HNC and other DRRD components, such as nuts, coffee, polyunsaturated/saturated fatty acids, trans-fat, GI, SSBs, and red meat and processed meat (Supplementary Tables S5–S11).

## Discussion

The present study provides evidence that a higher DRRD score is associated with a decreased incidence of HNC in a large US population of approximately 100,000 individuals, which was further confirmed by a series of sensitivity analyses. Moreover, subgroup analyses revealed that this inverse association was more pronounced in individuals who never smoked, indicating that adhering to the DRRD dietary pattern may benefit the population by reducing the risk of HNC, particularly among non-smokers. Additionally, among the nine components of the DRRD diet, it was found that high intake of cereal fiber and whole fruit was associated with a reduced risk of HNC, suggesting that promoting the intake of cereal fiber and whole fruit should be encouraged as part of the DRRD dietary pattern.

Over the past four decades, there has been a steady increase in the number of adults worldwide suffering from diabetes, growing from 108 million in 1980 to 463 million in 2019 (18). Diabetes is a systemic disease known to cause serious health complications, including kidney failure, peripheral arterial disease, infections, and cardiovascular disease (19), and it increases the risk of hypertension, obesity, and dyslipidemia (20). Additionally, increasing evidence suggests that individuals with diabetes are more susceptible to developing cancer (21). For instance, A meta-analysis of 36 researches revealed that people with diabetes had an adjusted odds ratio of 1.82 for pancreatic cancer compared to those without diabetes (22). Another meta-analysis of 20 studies concluded that diabetes is related to higher risks of both breast and colorectal cancer incidence as well as cancer-specific mortality (23). Four systematic reviews also found consistent results, indicating that diabetes elevates the risk of developing ovarian cancer (24–27). Specifically, a study conducted in an Asia population reported that diabetes is closely related to an enhanced risk of HNC (12). To tackle diabetes, the DRRD dietary pattern was developed in 2015 and has since gained popularity (28). Although originally developed for the prevention and control of diabetes, previous prospective studies have highlighted that following DRRD may reduce the incidence of pancreatic (29), liver (30), breast (31), and lung (9) cancers. To our knowledge, this study is the first to establish the association between adherence to the DRRD and a reduced risk of HNC. Therefore, the findings of this study may provide valuable dietary guidance for preventing HNC in the general population.

Several potential mechanisms may explain the association between DRRD and the reduced risk of HNC. Firstly, DRRD may lower the risk of HNC by reducing chronic inflammation, which has been linked to the development of tumors (32). DRRD dietary pattern recommends high intakes of fiber (33), nuts (34), coffee (35), polyunsaturated fat (36), and fruits (37), which are associated with lower inflammation levels. In contrast, DRRD recommends limiting the intake of high glycemic index foods (38), trans fatty acids (39), SBBs (40), red and processed meats (41), which are positively correlated with higher levels of inflammation. Importantly, it has been well established that higher adherence to DRRD was associated with lower levels of inflammation (42). Secondly, diabetes may increase the risk of obesity, which leads to the expression of tumor-susceptibility genes, tissue hypoxia, and a higher differentiation rate in adipose stromal cells, ultimately transforming normal cells into malignant tumors (43). Therefore, adhering to DRRD, which may reduce the risk of diabetes and obesity (28), could potentially lower the risk of oncogenesis. Thirdly, hyperinsulinemia and hyperglycemia are closely related to accelerated biological aging and the stimulation of cellular signaling pathways associated with growth factor-dependent cell proliferation and cancer development (9). Additionally, cancer cells consume large amounts of glucose when growing and proliferating (44). It has been reported that insulin resistance directly promotes carcinogenesis in diabetic individuals (45), and insulin-like growth factor-1 initiates and progresses tumor growth (46). Therefore, we speculate that the reduced risk of HNC may be attributed to the reduction of chronic inflammation, obesity, hyperinsulinemia, hyperglycemia, and insulin resistance related to DRRD dietary pattern.

Interestingly, our subgroup analyses revealed that the inverse association between the DRRD score and HNC was more pronounced in non-smokers. This observation may be linked to inflammation, which has been demonstrated to play a critical role in the development and progression of HNC (47). Studies have shown that smoking or an increase in smoking can lead to elevated levels of somatic inflammation (48, 49), whereas adherence to the DRRD can decrease these levels. Additionally, Ramo et al. (50) discovered that smokers are more likely to engage in multiple health-risk behaviors, including poor dietary habits and lack of physical activity. Therefore, we speculate that non-smokers may be more inclined to follow a healthy dietary pattern, such as the DRRD, to maintain good health.

Our study has some limitations. Firstly, the dietary information used to calculate the DRRD score was collected only once, which may not accurately reflect changes in dietary habits over time, leading to non-differential bias. Nevertheless, as adults’ dietary habits usually do not change significantly in nutritional epidemiology (51), this limitation may not be significant. Secondly, information on EBV and HPV infection was not obtained for each participant and could not be adjusted in the analysis due to data lacking, potentially affecting the results. However, since EBV and HPV infection status is unlikely to be specifically associated with dietary intake, it might not meet the properties of a confounder. Lastly, the study’s population was limited to individuals aged 55–74 years in the US, and therefore, caution should be exercised in applying the findings to other populations. Further research is needed to confirm the universality of our observed results in other populations.

In conclusion, our findings suggest that DRRD dietary pattern is associated with a reduced risk of HNC in a large US population, especially among non-smokers. These findings provide evidence that adherence to DRRD may be beneficial in preventing HNC.

## Data availability statement

The original contributions presented in the study are included in the article/Supplementary material, further inquiries can be directed to the corresponding authors.

## Ethics statement

The present study involved human participants who were reviewed and approved by the Institutional Review Board of the NCI and the ten PLCO trial screening centers. Written informed consent was obtained from all individuals in the PLCO study. This study has been approved by the NCI (approval number: PLCO-1140).

## Author contributions

LX designed the study. LP and LX request access to the original data. XW, CZ, and YX analyzed the data. XW, LP, HL, ZX, JW, HG, and YW assisted with statistical analysis. XW, CZ, and LX assisted in the interpretation of the results. XW, YX, CZ, and LX drafted the initial manuscript and revised the manuscript. All authors contributed to the article and approved the submitted version.

## Funding

This work was supported by Natural Science Foundation Project of Chongqing, Chongqing Science and Technology Commission, China [cstc2021jcyj-msxmX0153 (LP)], [cstc2021jcyj-msxmX0112 (YW)], and [CSTB2022NSCQ-MSX1005 (HG)], and Kuanren Talents Project of the Second Affiliated Hospital of Chongqing Medical University in China [kryc-yq-2110 (HG)].

## Conflict of interest

The authors declare that the research was conducted in the absence of any commercial or financial relationships that could be construed as a potential conflict of interest.

## Publisher’s note

All claims expressed in this article are solely those of the authors and do not necessarily represent those of their affiliated organizations, or those of the publisher, the editors and the reviewers. Any product that may be evaluated in this article, or claim that may be made by its manufacturer, is not guaranteed or endorsed by the publisher.

## Abbreviations

BQ: Baseline questionnaire
CIs: Confidence intervals
DHQ: Diet history questionnaire
DRRD: Diabetes risk reduction diet
EBV: Epstein-barr virus
FFQ: Food frequency questionnaire
GI: Glycemic index
HNC: Head and neck cancer
HPV: Human papillomavirus
HRs: Hazard ratios
NCI: National cancer institute
PLCO: Prostate lung colorectal and ovarian
SSBs: Sugar-sweetened beverages
