# Supplementary material for: Adherence to diabetes risk reduction diet and the risk of head and neck cancer: a prospective study of 101,755 American adults
Source: Front Nutr. 2023 Sep 22;10:1218632. doi: 10.3389/fnut.2023.1218632 (PMC10556244; doi:10.3389/fnut.2023.1218632)
Supplement: Supplementary file 1 [file Data_Sheet_1.PDF]

SUPPLEMENTARY MATERIALS

Adherence to Diabetes Risk Reduction Diet and the Risk of Head and Neck Cancer: A Prospective Study of 101,755 American Adults

Xia Wu[1], Linglong Peng[2], Haoyun Luo[2], Zhiquan Xu[2], Jijian Wang[2], Haitao Gu[2], Yaxu Wang[2], Yi Xiao[2*], Chaohua Zhang[2*] and Ling Xiang[3*]

[1] *Department of Health Management Centre, Chongqing General Hospital, Chongqing, China.*

[2] *Department of Gastrointestinal Surgery, The Second Affiliated Hospital of Chongqing Medical University, Chongqing, China.*

[3] *Department of Clinical Nutrition, The Second Affiliated Hospital of Chongqing Medical University, Chongqing, China.*

* Correspondence authors: Yi Xiao (1414054626@qq.com), Chaohua Zhang (742569563@qq.com), and Ling Xiang (306359@hospital.cqmu.edu.cn), The Second Affiliated Hospital of Chongqing Medical University, No.288 Tianwen Avenue, Nan'an District, Chongqing, China.

Supplementary Table 1. Criteria for determining diabetes risk reduction diet score

Points	Energy-adjusted dietary intakes of individual components								
	Cereal fiber (g/day)	Coffee (g/day)	Nuts (g/day)	Whole fruit (servings/day)	Ratio of polyunsaturated to saturated fat	Trans fat (g/day)	Glycemic Index from diet	Sugar-sweetened beverage (g/day)	Red and processed meat (g/day)
1	⩽7.21	⩽25.36	⩽0.43	⩽1.20	⩽0.55	⩾5.57	⩾56.20	⩾389.56	⩾18.87
2	7.22-9.65	25.37-441.72	0.44-1.39	1.21-1.93	0.56-0.67	4.00-5.56	54.40-56.19	181.26-389.55	9.86-18.86
3	9.66-12.16	441.73-1050.00	1.40-3.67	1.94-2.71	0.68-0.78	2.98-3.99	52.85-54.39	73.03-181.25	5.50-9.85
4	12.17-15.78	1050.01-1277.86	3.68-7.78	2.72-3.87	0.79-0.94	2.08-2.97	50.93-52.84	24.89-73.02	2.70-5.49
5	⩾15.79	⩾1277.87	⩾7.79	⩾3.87	⩾0.95	⩽2.07	⩽50.92	⩽24.88	⩽2.69

Supplementary Table 2. Distribution of covariates with missing data before and after imputation

Variable	Before imputation	After imputation	Number (%) with missing data
Race			
Non-Hispanic	97829 (98.37)	100136 (98.41)	2307 (2.27)
Hispanic	1619 (1.63)	1619 (1.59)	
Educational level			
College below	42937 (42.28)	42937 (42.20)	204 (0.20)
College and beyond	58614 (57.72)	58818 (57.80)	
Marital Status			
Live together	79633 (78.41)	79826 (78.45)	193 (0.19)
Live alone	21929 (21.59)	21929 (21.55)	
Smoking status			
Current	9401 (9.24)	9401 (9.24)	20 (0.02)
Former	43774 (43.03)	43774 (43.02)	
Never	48560 (47.73)	48580 (47.74)	
History of diabetes			
No	94411 (93.28)	94949 (93.31)	538 (0.53)
Yes	6806 (6.72)	6806 (6.69)	
Family history of NHC			
No	1447 (1.43)	1447 (1.42)	781 (0.77)
Yes	99527 (98.57)	100308 (98.58)	
Pack-years of smoking	17.83 ± 26.69	17.65 ± 26.59	1164 (1.14)
Body mass index (kg/m^2)	27.23 ± 4.82	27.22 ± 4.79	1348 (1.32)

Values are mean (standard deviation) or counts (percentage) as indicated.

Supplementary Table 3. Hazard ratios of the association of cereal fiber with the risk of HNC.

Quartiles of Cereal fiber (g/day)	Number of cases	Person-years	Incidence rate per 100 person-years (95% confidence interval)	Hazard ratio (95% confidence interval)		
				Unadjusted	Model 1 [a]	Model 2 [b]
Quartile 1 (≤7.86)	25480/88	222353.5	0.04 (0.032, 0.049)	1.000 (reference)	1.000 (reference)	1.000 (reference)
Quartile 2 (7.87-10.83)	25403/64	224547.4	0.029 (0.022, 0.036)	0.719 (0.521, 0.992)	0.684 (0.496, 0.944)	0.729 (0.521, 1.019)
Quartile 3 (10.84-14.67)	25448/70	226288.8	0.031 (0.024, 0.039)	0.780 (0.570, 1.067)	0.692 (0.505, 0.947)	0.737 (0.517, 1.051)
Quartile 4 (>14.67)	25424/57	226812.1	0.025 (0.019, 0.033)0	0.633 (0.454, 0.884)	0.506 (0.362, 0.707)	0.471 (0.301, 0.739)
P_{-trend}				**0.015**	**<0.001**	**0.002**

a: Adjusted for age, sex and race.
b: Adjusted for model 1 plus marital status, educational level, BMI, smoking status, pack-years of smoking, drinking status, alcohol consumption, history of diabetes, family history of HNC and energy from diet.

Supplementary Table 4. Hazard ratios of the association of whole fruit with the risk of HNC.

Quartiles of whole fruit intake (servings/day)	Number of participants/cases	Person-years	Incidence rate per 100 person-years (95% confidence interval)	Hazard ratio (95% confidence interval)		
				Unadjusted	Model 1[a]	Model 2[b]
Quartile 1 (≤1.40)	25665/106	223265.175	0.047 (0.039, 0.057)	1.00 (reference)	1.00 (reference)	1.00 (reference)
Quartile 2 (1.41-2.29)	25271/80	223535.066	0.036 (0.029, 0.045)	0.752 (0.562, 1.005)	0.785 (0.587, 1.051)	1.008 (0.748, 1.358)
Quartile 3 (2.30-3.51)	25501/55	227020.769	0.024 (0.019, 0.032)	0.508 (0.367, 0.704)	0.550 (0.396, 0.763)	0.777 (0.552, 1.093)
Quartile 4 (>3.51)	25318/38	226180.854	0.017 (0.012, 0.023)	0.352 (0.243, 0.510)	0.381 (0.262, 0.553)	0.555 (0.372, 0.829)
P_{-trend}				**<0.001**	**<0.001**	**0.002**

a: Adjusted for age, sex and race.
b: Adjusted for model 1 plus marital status, educational level, BMI, smoking status, pack-years of smoking, drinking status, alcohol consumption, history of diabetes, family history of HNC and energy from diet.

Supplementary Table 5. Hazard ratios of the association of nuts with the risk of HNC.

Quartiles of Nuts intake (g/day)	Number of participants/cases	Person-years	Incidence rate per 1000 person-years (95% confidence interval)	Hazard ratio (95% confidence interval)		
				Unadjusted	Model 1[a]	Model 2[b]
Quartile 1 (≤0.48)	25498/84	226402.519	0.037 (0.03, 0.046)	1.000 (reference)	1.000 (reference)	1.000 (reference)
Quartile 2 (0.49-1.79)	29105/83	257857.273	0.032 (0.026, 0.04)	0.868 (0.641, 1.176)	0.600 (0.441, 0.817)	0.637 (0.467, 0.868)
Quartile 3 (1.80-7.23)	24099/53	213549.692	0.025 (0.019, 0.032)	0.670 (0.475, 0.945)	0.579 (0.410, 0.818)	0.640 (0.451, 0.908)
Quartile 4 (>7.23)	23053/59	202192.381	0.029 (0.023, 0.038)	0.789 (0.566, 1.101)	0.521 (0.372, 0.731)	0.589 (0.413, 0.839)
P_{-trend}				0.306	0.0130	0.068

a: Adjusted for age, sex and race.

b: Adjusted for model 1 plus marital status, educational level, BMI, smoking status, pack-years of smoking, drinking status, alcohol consumption, history of diabetes, family history of HNC and energy from diet.

Supplementary Table 6. Hazard ratios of the association of coffee with the risk of HNC.

Quartiles of Coffee intake (g/day)	Number of participants/cases	Person-years	Incidence rate per 100 person-years (95% confidence interval)	Hazard ratio (95% confidence interval)		
				Unadjusted	Model 1[a]	Model 2[b]
Quartile 1 (≤152.07)	25640/49	226540.731	0.022 (0.016, 0.029)	1.00 (reference)	1.00 (reference)	1.00 (reference)
Quartile 2 (152.08-887.07)	26998/56	238831.838	0.023 (0.018, 0.03)	1.084 (0.739, 1.591)	1.073 (0.731, 1.574)	0.944 (0.640, 1.395)
Quartile 3 (887.08-1277.87)	34279/113	303903.441	0.037 (0.031, 0.045)	1.719 (1.229, 2.404)	1.422 (1.016, 1.992)	1.064 (0.750, 1.509)
Quartile 4 (>1277.87)	14838/61	130725.854	0.047 (0.036, 0.06)	2.159 (1.482, 3.144)	1.757 (1.204, 2.564)	0.943 (0.632, 1.408)
P_{-trend}				<0.001	0.001	0.876

a: Adjusted for age, sex and race.

b: Adjusted for model 1 plus marital status, educational level, BMI, family history of HNC, smoking status, pack-years of smoking, drinking status, alcohol consumption, history of diabetes, family history of HNC and energy from diet.

Supplementary Table 7. Hazard ratios of the association of ratio of polyunsaturated to saturated fat with the risk of HNC.

Quartiles of ratio of polyunsaturated to saturated fat	Number of participants/cases	Person-years	Incidence rate per 1000 person-years (95% confidence interval)	Hazard ratio (95% confidence interval)		
				Unadjusted	Model 1[a]	Model 2[b]
Quartile 1 (≤0.586)	25439/100	221968.112	0.045 (0.037, 0.055)	1.000 (reference)	1.000 (reference)	1.000 (reference)
Quartile 2 (0.587-0.727)	25439/71	224971.187	0.032 (0.025, 0.04)	0.700 (0.516, 0.948)	0.755 (0.557, 1.024)	0.845 (0.622, 1.147)
Quartile 3 (0.728-0.896)	25438/64	225790.554	0.028 (0.022, 0.036)	0.628 (0.459, 0.860)	0.736 (0.537, 1.009)	0.882 (0.641, 1.213)
Quartile 4 (>0.896)	25439/44	227272.011	0.019 (0.014, 0.026)	0.429 (0.301, 0.612)	0.548 (0.384, 0.784)	0.684 (0.475, 0.985)
P_{-trend}				<0.001	0.001	0.054

a: Adjusted for age, sex and race.
b: Adjusted for model 1 plus marital status, educational level, BMI, smoking status, pack-years of smoking, drinking status, alcohol consumption, history of diabetes, family history of HNC and energy from diet.

Supplementary Table 8. Hazard ratios of the association of trans fat with the risk of HNC.

Quartiles of trans fat consumption (g/day)	Number of participants/cases	Person-years	Incidence rate per 100 person-years (95% confidence interval)	Hazard ratio (95% confidence interval)		
				Unadjusted	Model 1[a]	Model 2[b]
Quartile 1 (≤2.30)	25659/51	227761.799	0.022 (0.017, 0.029)	1.000 (reference)	1.000 (reference)	1.000 (reference)
Quartile 2 (2.31-3.45)	25384/55	225030.333	0.024 (0.019, 0.032)	1.092 (0.746, 1.598)	0.960 (0.655, 1.407)	1.011 (0.684, 1.494)
Quartile 3 (3.46-5.08)	25354/73	224540.268	0.033 (0.026, 0.041)	1.452 (1.015, 2.077)	1.121 (0.781, 1.610)	1.191 (0.802, 1.768)
Quartile 4 (>5.08)	25358/100	222669.465	0.045 (0.037, 0.055)	2.007 (1.433, 2.812)	1.312 (0.927, 1.857)	1.500 (0.937, 2.402)
P_{-trend}				<0.001	0.05	0.053

a: Adjusted for age, sex and race.
b: Adjusted for model 1 plus marital status, educational level, BMI, smoking status, pack-years of smoking, drinking status, alcohol consumption, history of diabetes, family history of HNC and energy from diet.

Supplementary Table 9. Hazard ratios of the association of glycemic index with the risk of HNC.

Quartiles of Glycemic Index	Number of participants/cases	Person-years	Incidence rate per 1000 person-years (95% confidence interval)	Hazard ratio (95% confidence interval)		
				Unadjusted	Model 1[a]	Model 2[b]
Quartile 1 (≤51.48)	25464/68	223710.587	0.03 (0.024, 0.039)	1.000 (reference)	1.000 (reference)	1.000 (reference)
Quartile 2 (51.49-53.62)	25488/67	225602.146	0.03 (0.023, 0.038)	0.976 (0.696, 1.367)	0.909 (0.648, 1.274)	1.034 (0.734, 1.455)
Quartile 3 (53.63-55.68)	25388/68	225552.534	0.03 (0.024, 0.038)	0.990 (0.707, 1.385)	0.880 (0.629, 1.233)	0.993 (0.705, 1.399)
Quartile 4 (>55.68)	25415/76	225136.597	0.034 (0.027, 0.042)	1.108 (0.799, 1.537)	0.962 (0.693, 1.335)	0.993 (0.709, 1.392)
$P_{\text{-trend}}$				0.536	0.799	0.921

a: Adjusted for age, sex and race.
b: Adjusted for model 1 plus marital status, educational level, BMI, smoking status, pack-years of smoking, drinking status, alcohol consumption, history of diabetes, family history of HNC and energy from diet.

Supplementary Table 10. Hazard ratios of the association of sugar-sweetened beverage with the risk of HNC.

Quartiles of Glycemic Index	Number of participants/cases	Person-years	Incidence rate per 1000 person-years (95% confidence interval)	Hazard ratio (95% confidence interval)		
				Unadjusted	Model 1[a]	Model 2[b]
Quartile 1 (≤31.950)	25481/74	225314.908	0.033 (0.026, 0.041)	1.000 (reference)	1.000 (reference)	1.000 (reference)
Quartile 2 (31.951-110.220)	25400/55	226088.753	0.024 (0.019, 0.032)	0.740 (0.522, 1.049)	0.669 (0.472, 0.950)	0.767 (0.540, 1.091)
Quartile 3 (110.221-318.195)	25435/64	225365.862	0.028 (0.022, 0.036)	0.864 (0.619, 1.208)	0.743 (0.530, 1.041)	0.884 (0.628, 1.244)
Quartile 4 (>318.195)	25439/86	223232.342	0.039 (0.031, 0.048)	1.174 (0.860, 1.602)	1.006 (0.732, 1.383)	1.140 (0.821, 1.583)
$P_{\text{-trend}}$				0.036	0.167	0.087

a: Adjusted for age, sex and race.
b: Adjusted for model 1 plus marital status, educational level, BMI, smoking status, pack-years of smoking, drinking status, alcohol consumption, history of diabetes, family history of HNC and energy from diet.

Supplementary Table 11. Hazard ratios of the association of red and processed meat with the risk of HNC.

Quartiles of red meat and processed meat intake (g/day)	Number of participants/cases	Person-years	Incidence rate per 100 person-years (95% confidence interval)	Hazard ratio (95% confidence interval)		
				Unadjusted	Model 1[a]	Model 2[b]
Quartile 1 (≤ 3.32)	25462/33	227711.387	0.014 (0.01, 0.02)	1.000 (reference)	1.000 (reference)	1.000 (reference)
Quartile 2 (3.33-7.41)	25444/63	225860.591	0.028 (0.022, 0.036)	1.926 (1.264, 2.935)	1.654 (1.084, 2.524)	1.552 (1.014, 2.375)
Quartile 3 (7.42-15.79)	25425/84	224136.485	0.037 (0.03, 0.046)	2.590 (1.732, 3.875)	1.860 (1.236, 2.800)	1.650 (1.086, 2.508)
Quartile 4 (≥15.80)	25424/99	222293.402	0.045 (0.037, 0.054)	3.080 (2.077, 4.567)	1.848 (1.230, 2.775)	1.505 (0.972, 2.331)
$P_{\text{-trend}}$				<0.001	0.042	0.48

a: Adjusted for age, sex and race.

b: Adjusted for model 1 plus marital status, educational level, BMI, smoking status, pack-years of smoking, drinking status, alcohol consumption, history of diabetes, family history of HNC and energy from diet.
